# A Comprehensive Characterization of Patients with Spinal Cord Neurosarcoidosis: A Single Center Cross-Sectional Study of Clinical Outcomes

**DOI:** 10.3390/jcm13175069

**Published:** 2024-08-27

**Authors:** Rami Al-Hader, Justin Nofar, Ahmed Mohamedelkhair, Muhammad Affan, Lonni R. Schultz, Mirela Cerghet

**Affiliations:** 1 Department of Neurology, Henry Ford Health, Detroit, MI 48202, USA; ramihader@hotmail.com (R.A.-H.);; 2 Department of Neurology, University of Minnesota, Minneapolis, MN 55455, USA; 3 Department of Public Health Sciences, Henry Ford Health, Detroit, MI 48202, USA; 4 School of Medicine, Wayne State University, Detroit, MI 48201, USA

**Keywords:** neurosarcoidosis, spinal cord, sarcoidosis, immunosuppressive agents

## Abstract

**Background/Objective:** To describe the clinical features and radiological outcomes of patients with spinal cord neurosarcoidosis, treatments, and long-term follow-up for this rare disorder. **Methods:** A cross-sectional, retrospective medical chart review was performed for all patients with spinal cord neurosarcoidosis treated at a single center between 01/1995 and 12/2020. Radiological imaging, laboratory test results, the type of immunosuppressive therapy, and function test scores were reviewed. **Results:** We assessed 39 patients with spinal cord neurosarcoidosis (23 men, 16 women, mean age at presentation 46.4 years, SD 10.2 years). The mean (SD) duration of spinal cord neurosarcoidosis at data abstraction was 9.8 (6.3) years. There were 24 patients (62%) with extensive intramedullary lesions, 8 (21%) with multiple patchy intramedullary lesions, 12 (31%) with leptomeningeal involvement, and 7 (18%) with nerve root enhancement. The cervical spine was the most commonly affected region in 33 patients (85%). The most common presenting symptoms were paresthesia/neuropathic pain in 20 (51%) and weakness of extremities in 15 (38%) patients. Most patients (*n* = 37; 95%) had been treated with corticosteroids at symptom onset, and methotrexate was the most used immunosuppressive therapy (*n* = 19; 49%). Of 34 patients with follow-up magnetic resonance imaging (MRI) available, the median time to improvement per MRI was 10.8 months (95% CI, 6.1–17.0 months). Of 31 patients with MRI enhancement at presentation, 18 (58%) had complete enhancement resolution at follow-up, with a median time to resolution of 51.8 months (95% CI, 24.9–83.4 months). Patients had significantly lower pyramidal (*p* = 0.004) and sensory functional (*p* = 0.031) systems scores from presentation to the last clinic visit. **Conclusions:** Because spinal cord neurosarcoidosis is challenging to diagnose and no set treatment guidelines exist, clarifying patients’ clinical parameters and responses to various treatments is needed to improve timely and efficient care. The incidence of spinal cord involvement in sarcoidosis in our cohort was higher than intracranial involvement and most patients had a long extensive intramedullary lesion. We also observed that most patients with spinal cord neurosarcoidosis improved clinically and radiologically after treatment; however, the resolution of MRI enhancement after immunosuppressive therapy may take years. Prospective studies of neurosarcoidosis will be crucial to address questions about effective treatment and long-term prognosis.

## 1. Introduction

Sarcoidosis is a multisystem granulomatous disease of unclear etiology, and can affect almost any organ or tissue, though most commonly affects the lungs, heart, lymph nodes, and skin, with a peak incidence in individuals 20 to 39 years old [1]. Up to 25% of the autopsies of patients who had sarcoidosis reveal central nervous system (CNS) involvement [2]. In up to 74% of patients diagnosed with neurosarcoidosis, neurological symptoms precede the diagnosis of sarcoidosis [3]. The most commonly reported neurosarcoidosis presentations are cranial nerve palsy [4] and optic neuritis followed by myelitis [5]. Notably, the diagnosis of neurosarcoidosis is challenging, and no simple definitive tests are available. Confirmatory biopsy remains the most reliable standard, but this is an invasive test of the CNS and is not the preferred approach. Cerebrospinal fluid (CSF) analysis may indicate the presence of inflammation, with the most common finding being a non-specific elevation in protein level [6]. Although the angiotensin-converting enzyme (ACE) level in CSF has been proposed as a potential biomarker, it is relatively nondiagnostic and of unclear value for neurosarcoidosis [7]. Magnetic resonance imaging (MRI) with gadolinium helps detect nervous system involvement; however, MRI is neither sensitive nor specific. 

The diagnostic criteria for neurosarcoidosis were proposed by Zajicek in 1999 and comprised mainly histological and other supportive clinical, laboratory and imaging data that categorize diagnoses as definite, probable, or possible neurosarcoidosis [6]. The diagnostic criteria were updated in 2018 by the Neurosarcoidosis Consortium Consensus Group [8]. A definite diagnosis of neurosarcoidosis requires clinical presentation and diagnostic evaluation to suggest neurosarcoidosis, with nervous system pathology consistent with neurosarcoidosis; Type A neurosarcoidosis includes evidence of extraneural sarcoidosis, whereas Type B has no evidence of extraneural sarcoidosis and is considered isolated to the CNS. A probable diagnosis includes a clinical and diagnostic evaluation suggestive of neurosarcoidosis, with a pathologic confirmation of systemic granulomatous disease consistent with sarcoidosis. Possible diagnoses included clinical and diagnostic evaluation suggestive of neurosarcoidosis without evidence of a pathologic confirmation of granulomatous disease. 

Approximately 17% of patients with neurosarcoidosis have no other organ involvement [3], and a diagnosis is difficult to reach without a nervous system biopsy. Spinal cord involvement has been reported in 18%–26% of patients with neurosarcoidosis [9,10], and several mechanisms have been proposed for sarcoidosis affecting the spinal cord, including the immune infiltration of the leptomeninges, spinal parenchyma, and extradural space or extraspinal tissue [9], leading to symptoms related to spinal cord dysfunction or compression. 

Clinical and radiological findings for patients with spinal cord neurosarcoidosis have been reported in a few studies; however, the best treatment options and the progression of the disease are poorly understood. Our current knowledge regarding the treatment and prognosis of neurosarcoidosis comes only from case series. Notably, no FDA-approved medications for neurosarcoidosis are currently available, and no placebo-controlled clinical trials in neurosarcoidosis have been carried out. Therefore, we conducted a single-center, retrospective cross-sectional study of patients who had neurosarcoidosis with spinal cord involvement. We aimed to define the clinical characteristics of this patient population while being treated with immunosuppressive agents, and explore patients’ clinical and radiological outcomes over time. An improved understanding of how patients clinically manifest spinal cord neurosarcoidosis and respond to various therapeutic strategies is greatly needed to refine clinical guidelines and design robust clinical trials to treat this rare disorder. 

## 2. Materials and Methods

### 2.1. Study Design and Data Collection

This was a retrospective, cross-sectional medical chart review study of patients with spinal cord neurosarcoidosis treated at a single institution in Detroit, MI, USA between January 1995 and December 2020. A computer search strategy was used to identify all patients in the medical record at our institution with ICD-9 code neurosarcoidosis (135). A database of 121 patients with neurosarcoidosis was created [10], and patients with spinal cord neurosarcoidosis were identified. Neurosarcoidosis diagnosis was based on the diagnostic criteria updated in 2018 (definite, probable, and possible) [8]. The clinical data extracted included age at diagnosis, sex, race as self-reported in the medical record, first neurological symptoms, extra-CNS sarcoidosis involvement, laboratory test results, biopsy results (where available), MRI findings, disease course, treatment regimen, and response to treatment. For the response to treatment, follow-up clinical notes until 12/2020 were reviewed for reported improvement of symptoms, calculating the pyramidal functional system score and sensory functional system score, part of the expanded disability status scale (EDSS) [11], and modified Rankin score (mRS) [12]. The follow-up MRI results of patients who had been treated were reviewed to assess stability or improvement in lesion size and enhancement. 

### 2.2. Statistical Analysis

Descriptive statistics were used to characterize patients with spinal cord neurosarcoidosis. Fisher’s exact test was used to assess the association of sarcoid involvement, type of lesion, and MRI brain lesion with laboratory and CSF data, and enhancement resolution and clinical improvement. Paired *t*-tests were used to compare pyramidal and sensory function scores between the presentation and the last visit. Kaplan–Meier methods were used to estimate the course of MRI improvement, gadolinium contrast (GAD) resolutions over time, and the median time to these events. The significance testing level was set at 0.05. SAS version 9.4 was used for all data analyses. The institutional review board approved this study (approval code # 6305; approved on 21 July 2010).

## 3. Patient Information

### 3.1. Study Population 

Of 110 patients with neurosarcoidosis, 39 (35.4%) had been diagnosed with spinal cord neurosarcoidosis and were included in the study: 23 (59%) were male, 16 (49%) were female, 26 (69%) were Black, and 11 (28%) were White. The mean (SD) age at presentation was 46.4 (10.2) years, while the mean (SD) duration of spinal cord neurosarcoidosis at data abstraction was 9.8 (6.3) years. Of the 39 patients, 7 (18%) had a CNS biopsy fulfilling the criteria for definitive neurosarcoidosis, while 26 (72%) had a probable diagnosis, and 4 (10%) had a possible diagnosis. (Table 1). 

### 3.2. Clinical Presentation

The two most common symptoms at presentation were paresthesia/neuropathic pain in 20 patients (51%) and weakness of extremities in 15 (38%). Fourteen patients (36%) had a previous diagnosis of sarcoidosis with multiorgan involvement outside the CNS, and 7 (18%) had only brain involvement before spinal cord involvement. There were 32 patients (84%) who had associated degenerative disease of the spine, which did not appear to be associated with age. Many patients had sarcoidosis outside the spinal cord, the most common sites being 24 (62%) in the lungs, 19 (49%) in the brain, 10 (26%) in the lymph nodes, and 8 (21%) in the cranial nerves. Twenty-two patients (56%) with spinal cord symptoms had no prior diagnosis of sarcoidosis at presentation (Table 1).

### 3.3. Diagnostic Assessment

**Figure 1:**
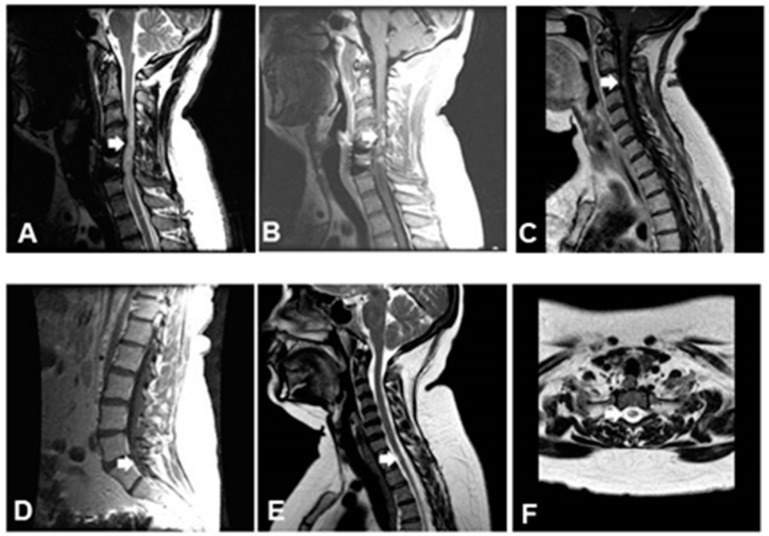
Magnetic resonance imaging (MRI) of patients with spinal cord neurosarcoidosis. Arrows show (**A**) a long extensive T2-weighted lesion; (**B**) enhancement of lesion on T1-weighted imaging with gadolinium; (**C**) curvilinear foci of leptomeningeal enhancement on T1-weighted imaging with gadolinium; (**D**) enhancement on T1-weighted imaging with gadolinium in cauda equine; (**E**) patchy T2 signal on sagittal spine image and (**F**) axial spine.

All 39 patients had spinal cord MRI results at presentation, and 36 also had brain MRI results. Different abnormalities were noted in the spine MRI results. They were categorized as long extensive intramedullary lesions (if T2 hyperintense lesion was spanning over three or more vertebrae), patchy lesions (T2 hyperintense lesion was spanning over two or less vertebrae), leptomeningeal nodular enhancement, and nerve roots enhancement (Figure 1).

**Figure 2:**
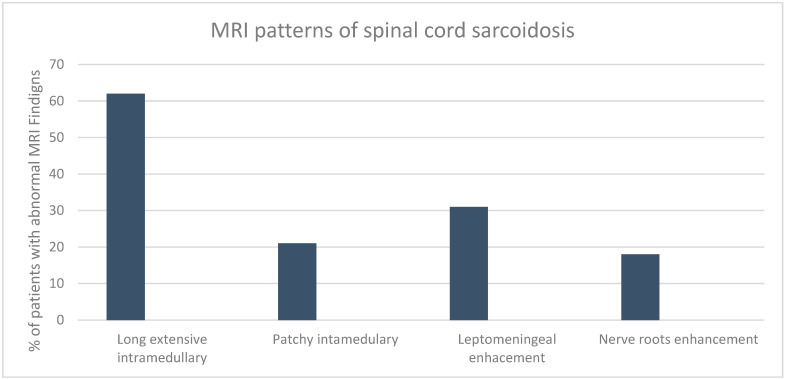
Characteristic imaging findings of spinal cord neurosarcoidosis at presentation.

Of the 39 patients, 24 (62%) had extensive intramedullary lesions, 8 (21%) had one or multiple patchy intramedullary lesions, 12 (31%) had leptomeningeal involvement, and 7 (18%) had nerve root enhancement, with lesions reported separately (Figure 2). Overall, the cervical spine was the most commonly affected region (*n* = 33; 85%), followed by the thoracic spine (*n* = 15; 38%) and the lumbar spine/cauda equina (*n* = 6; 15%). In some patients, extensive lesions spanned cervical and thoracic segments (*n* = 9; 23%), and almost all long extensive intramedullary lesions were enhanced with gadolinium (21 of the 23 patients with gadolinium data available). Of the 36 patients who had brain MRI, abnormal findings were found in 22 (61%) patients.

The results of the serum and CSF laboratory tests are listed in Table 2. Not all patients had undergone all tests. ACE in the blood was elevated in 17 of 35 patients (49%), but only 6 of 25 patients tested (24%) had ACE in the CSF. Most of the 16 patients with a serum C-reactive protein measurement had elevated levels (*n* = 13; 81%). Of patients for whom CSF measurements were carried out, 23/34 (68%) had elevated protein levels, 20/28 (71%) had elevated IgG, and 25/34 (74%) had elevated white blood cell counts.

Patients with long extensive lesions were significantly less likely to have elevated serum calcium levels than those without extensive lesions (9% vs 46%; *p* = 0.032). 

## 4. Clinical Findings

Of the 39 patients with spinal cord neurosarcoidosis, 13 (33%) had a monophasic disease, 15 (38%) had relapses (mostly while off treatment), and 10 (26%) had disease progression. One patient had no data available on their disease course. Almost all the patients (*n* = 37; 95%) were treated with oral or intravenous corticosteroids at the onset of symptoms, followed by maintenance with oral steroids for different time intervals. As patients required different immunosuppressive agents over time due to side effects or lack of response, methotrexate was the most used agent (*n* = 19; 49%), followed by azathioprine (*n* = 12; 31%) and mycophenolate mofetil (*n* = 7; 18%).

**Figure 3:**
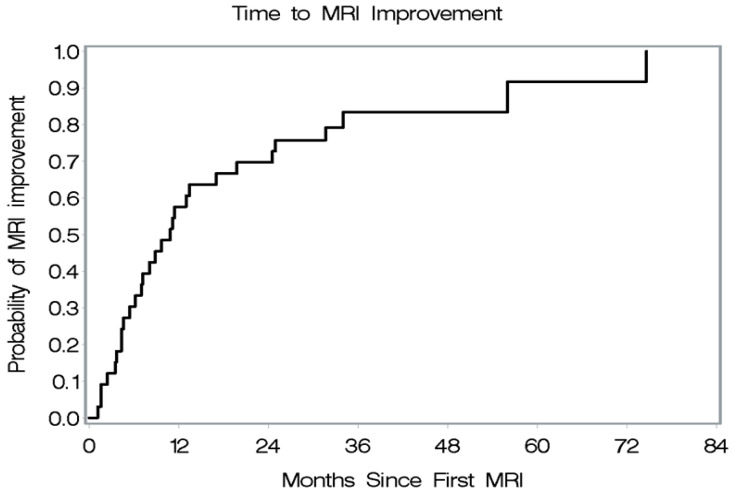
Time to MRI improvement with treatment.

**Figure 4:**
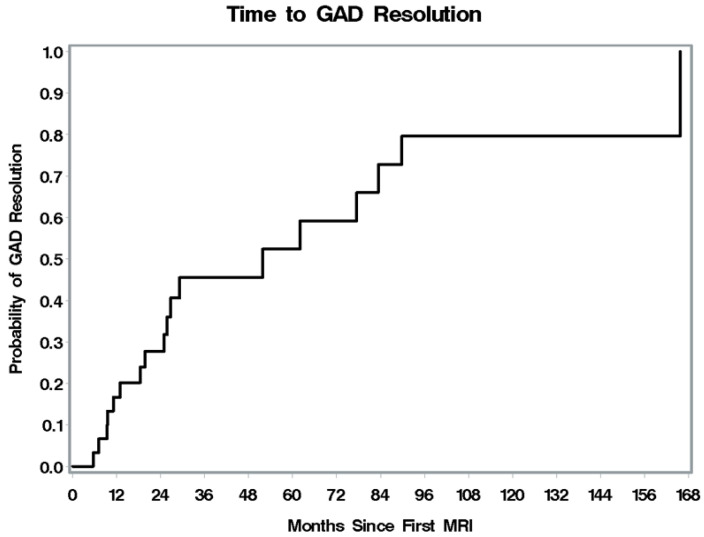
Time to GAD resolution with treatment of sarcoidosis. (Abbreviation: GAD, Gadolinium).

Follow-up MRI results were available for 34 patients (1 patient had no MRI; 4 patients had only one MRI). Of the 34 patients with follow-up MRI, 29 (85%) had documented improvement during follow-up, with a median time of improvement after MRI of 10.8 months (95% CI, 6.1 to 17 months) (Figure 3). Thirty-one patients had enhancement on MRI at presentation, and 18 (58%) had complete enhancement resolution during follow-up, with a median time for resolution of GAD enhancement of 51.8 months (95% CI, 24.9 to 83.4 months) (Figure 4). The specific treatments patients received during their follow-up MRI outcomes are in Table 3.

Clinical follow-up notes were available for 38 patients. While 25 patients had improvement in their initial symptoms and neurological examination, 10 remained stable, and 3 reported worsening symptoms. mRS was obtained by reviewing initial and final clinic notes. Of the 26 patients with both scores, the mRS score was improved at the final visit in 22 patients, with the majority improving by one point, whereas 1 patient had stable mRS and 3 had an increase in mRS. The pyramidal and sensory functional systems scores part of EDSS at the last visit were significantly lower than at the presentation. While the mean difference in sensory function score at the last visit was 0.36 points lower (*p* = 0.031), the mean difference in pyramidal function score at the last visit was 0.61 lower (*p* = 0.004). (Table 4).

## 5. Discussion

In this cross-sectional study, we assessed the clinical landscape of spinal cord neurosarcoidosis, highlighting the most common imaging and laboratory findings, disease course, and treatment response for a population of 39 patients. Spinal cord neurosarcoidosis is a rare [3,9] sarcoidosis manifestation, and our current knowledge about clinical presentation and treatment is based mainly on case reports. Diagnosing neurosarcoidosis can be quite challenging, and this condition is often initially misdiagnosed since there are no specific clinical, radiological, or laboratory findings. The most definitive diagnostic test is spinal cord biopsy, but it is not commonly performed due to the potential for serious complications related to the procedure.

In our study, about one-third of 110 patients with neurosarcoidosis had spinal cord involvement. Spinal cord neurosarcoidosis has been reported in previous studies, predominantly in males with a median age of over 40 years [9,10], similar to our study. A large proportion (69%) of our patients were Black, a higher percentage than reported in other studies [13]. Our patient distribution may be more related to the demographic distribution of our patient population. We also observed that paresthesia/neuropathic pain was the most common symptom at presentation, similar to other studies [13,14]. However, other studies have reported higher levels of back pain [3] and motor deficit [9] than we observed. 

Upon thorough examination, most patients with spinal cord neurosarcoidosis show evidence of sarcoidosis outside the CNS. Obtaining a biopsy from outside the CNS helps diagnose neurosarcoidosis; however, problematic cases remain for those with isolated neurosarcoidosis. Some clues on the typical radiological presentation of spinal cord neurosarcoidosis can be extracted from several case reports [9,13]. Intramedullary lesions are the most reported findings in patients with spinal cord neurosarcoidosis, and long extensive spinal lesions have been reported [9,10,13]. In our cohort, long extensive lesions were commonly found; notably, nine patients had spinal lesions spanning the cervical and thoracic regions. Degenerative spinal disease was commonly seen in over 80%. Cervical spondylosis has been reported in patients with sarcoidosis myelopathy [13,15,16], and it has been hypothesized that chronic compression of the spinal cord leads to the disruption of the blood-spinal cord barrier, as a common mechanism related to the perivascular spreading of inflammation [13,17]

CSF analysis is currently used in the diagnostic criteria for neurosarcoidosis, which were last updated in 2018 by the Neurosarcoidosis Consortium Consensus Group [8]. However, CSF analysis for assessing spinal cord neurosarcoidosis remains variable and is often non-specific. Regardless, the presence of elevated protein, elevated IgG index, oligoclonal bands, elevated white blood cell count, and low glucose can provide evidence of active inflammation, which we observed in our cohort, particularly elevated protein and white cell count. Contrary to common belief, ACE level in CSF has poor sensitivity and specificity for neurosarcoidosis [7,18] and may not have a significant added diagnostic value. 

Many questions remain regarding the best treatment strategies for patients with sarcoidosis, including spinal cord neurosarcoidosis. Currently, there are no FDA-approved treatments for this condition, and no robust clinical trials for neurosarcoidosis have been carried out. Thus, treatment approaches for neurosarcoidosis are mainly based on findings from case reports, strategies for treating pulmonary sarcoidosis that primarily include immunosuppressive agents. Typically, patients are started on corticosteroids, and then a steroid-sparing immunosuppressant drug is added while lowering the corticosteroid dose to avoid long-term side effects from corticosteroids. However, the duration of treatment and prognosis for this rare and variable manifestation of sarcoidosis remain to be determined. Joubert et al. conducted one of the largest retrospective analyses, which showed that immunosuppressive therapy with intravenous cyclophosphamide, methotrexate, or infliximab were associated with lower neurosarcoidosis relapse rates [19]. Methotrexate was compared to mycophenolate mofetil for treating neurosarcoidosis in a small study, which showed that methotrexate therapy led to increased survival time without relapse [20]. Infliximab has been reported as an efficacious therapy for refractory neurosarcoidosis in one study [21]; however, 50% of patients had a relapse in the first year of discontinuation [22].

Our patient population used immunosuppressive therapies with or without corticosteroids after initial corticosteroid treatment for an acute presentation. Methotrexate and azathioprine were the most commonly used agents, and only a few patients were treated with infliximab, which had only become available in recent years. Our patients showed significant clinical improvement after receiving various immunosuppressive agents, as measured with the pyramidal and the sensory functional scores of EDSS between the initial presentation and the last clinic visit. Furthermore, as noted in the follow-up MRI studies, many patients had a significant response and improvement. However, the resolution of contrast enhancement seen on MRI findings was delayed, with a median resolution time of 51.8 months. This observation supports the need for long-term immunosuppressive therapy for patients with neurosarcoidosis, as previously reported [22]. For this reason, steroid-sparing immunosuppressive therapies should be considered as soon as a diagnosis of spinal cord neurosarcoidosis is reached.

### Limitations

Our study had several limitations. First, it was a retrospective study and is subject to potential selection biases. Also, the assessment of patients’ responses to treatment was not standardized, and self-reported improvement in symptoms and a review of clinical neurological examination notes were used. mRS was also calculated; however, this is not a sensitive test, and other disability scales used to assess spinal cord disorders might be appropriate in a prospective study. Moreover, the choice of treatment was at the physicians’ discretion, and there was no treatment stratification, making the comparison between immunosuppressive therapeutic approaches difficult. A good clinical response was attributed to the effect of the immunosuppressive therapy used at the time of the follow-up MRI. Prospective studies of neurosarcoidosis will be crucial to address questions about effective treatment, duration of treatment, and disease course. 

## 6. Conclusions

Accurately diagnosing and effectively treating patients with spinal cord neurosarcoidosis is challenging. Spinal cord inflammation can be the initial presentation of sarcoidosis, and clinical and radiological findings can provide clues, raising the suspicion for neurosarcoidosis for a timely evaluation and treatment. A third or less of the patients had extra nervous system sarcoidosis and/or intracranial involvement of sarcoidosis at presentation. Cervical spine was the most affected by sarcoidosis in our cohort, and spinal cord radiological findings were diverse, with more than half of patients having a long extensive intramedullary abnormality. We observed that patients with spinal cord neurosarcoidosis had improved radiological findings and clinical parameters after immunosuppressive treatment, suggesting that imaging guidelines for this rare sarcoidosis manifestation may be clinically valuable. Also, MRI enhancement resolution in patients with spinal cord neurosarcoidosis may not occur until after several years of immunosuppressive therapy, which is longer than required for other spinal neuroimmunological conditions. Our current knowledge about the best treatments for neurosarcoidosis and the probable prognosis for this disease are limited, with no FDA-approved therapies or robust clinical trial data available; therefore, a more thorough characterization of the clinical landscape and treatment outcomes for patients with spinal cord neurosarcoidosis, as shown in this study, should aid in the development of guidelines and design of robust clinical studies.

## Figures and Tables

**Table 1:** Demographic and clinical characteristics of patients with spinal cord neurosarcoidosis.

Characteristic	Result *n* (%) *n* = (39)
Age at diagnosis, years, mean (SD)	46.4 (10.2)
Sex	
Female	16/39 (41)
Male	23/39 (59)
Race	
Black	27/39 (69)
White	11/39 (28)
Other	1/39 (3)
Duration of spinal cord neurosarcoidosis, years, mean (SD)	9.8 (6.3)
Diagnosis	
Definite	7/39 (18)
Probable	28/39 (72)
Possible	4/39 (10)
Symptoms at presentation	
Balance problems	1/39 (3)
Headache	1/39 (3)
Loss of consciousness	1/39 (3)
Paresthesia/neuropathic pain	20/39 (51)
Spasticity	1/39 (3)
Weakness of extremities	15/39 (38)
Degenerative disease of the spine	32/38 (84)
Sarcoidosis before spinal cord involvement	
CNS involvement only	7/39 (18)
Extra CNS involvement	14/39 (36)
Other sarcoidosis involvement (before and after spinal cord diagnosis), *n* (%)	
Abdominal mass	1/39 (3)
Bone	2/39 (5)
Brain	19/39 (49)
Cranial nerve	8/39 (21)
Ear	1/39 (3)
Joints	2/39 (5)
Liver	3/39 (8)
Lung	24/39 (62)
Lymphadenopathy/lymph nodes	10/39 (26)
Optic neuritis	1/39 (3)
PNS	2/39 (5)
Skin	5/39 (13)
Uveitis	2/39 (5)

Abbreviations: CNS, central nervous system; SD, standard deviation; PNS, peripheral nervous system.

**Table 2:** Blood and spinal fluid laboratory test results for patients with spinal cord neurosarcoidosis.

Clinical Finding	*n* (%) (*n* = 39)
Laboratory tests	
25-hydroxy vitamin D low	19/26 (73)
25-hydroxy vitamin D elevated	4/15 (27)
C-Reactive Protein elevated	13/16 (81)
Calcium elevated	8/35 (23)
Serum ACE elevated	17/35 (49)
Cerebrospinal fluid	
ACE elevated	6/25 (24)
Glucose low	6/31 (19)
IgG elevated	20/28 (71)
IgG index elevated	10/26 (38)
Oligoclonal bands	6/27 (22)
Protein elevated	23/34 (68)
WBC elevated	25/34 (74)

Abbreviations: ACE, angiotensin-converting enzyme; WBC, white blood cells.

**Table 3:** Initial and follow-up drug therapies for patients with spinal cord neurosarcoidosis.

	Therapies Received, *n* (%) (*n* = 39)		
Therapeutic Reagent	Patients Received Treatment *n* (%)	Patients with Lesion Reduction Observed on MRI While on Treatment	Patients with GAD Resolution While on Treatment
Azathioprine	12 (31)	5	3
Corticosteroids	37 (95)	22	11
Hydroxychloroquine	6 (15)	1	1
Infliximab	5 (13)	1	1
Methotrexate	19 (49)	6	5
Mycophenolate mofetil	7 (18)	4	2

Abbreviations: GAD, gadolinium; MRI, magnetic resonance imaging.

**Table 4:** Pyramidal function scores and sensory functional systems scores over time for patients with spinal cord neurosarcoidosis.

	Time Score Taken, Mean (SD)			
	Presentation	Last Visit	Difference	*p*-Value
Pyramidal function score (*n* = 33)	2.48 (1.23)	1.88 (1.34)	0.61 (1.14)	0.004
Sensory function score (*n* = 30)	1.63 (0.96)	1.27 (0.94)	0.36 (0.89)	0.031

Abbreviations: SD, standard deviation.

## Data Availability

The original contributions presented in the study are included in the article, further inquiries can be directed to the corresponding author.
